# Stress-Inducible Caspase Substrate TRB3 Promotes Nuclear Translocation of Procaspase-3

**DOI:** 10.1371/journal.pone.0042721

**Published:** 2012-08-09

**Authors:** Kouhei Shimizu, Shoukichi Takahama, Yaeta Endo, Tatsuya Sawasaki

**Affiliations:** 1 The Cell-Free Science and Technology Research Center, Ehime University, Matsuyama, Japan; 2 The Venture Business Laboratory, Ehime University, Matsuyama, Japan; 3 Proteo-Medicine Research Center, Ehime University, Toon, Japan; 4 RIKEN Systems and Structural Biology Center, Yokohama, Japan; Roswell Park Cancer Institute, United States of America

## Abstract

Pseudokinase TRB3 is a stress-inducible nuclear protein, which has recently been shown to be involved in ER stress-induced apoptosis. However, it remains unclear how TRB3 contributes to the process. We recently demonstrated that TRB3 was cleaved by caspase-3 (CASP3) *in vitro* and also in apoptosis-induced cells. Thus, we investigate the role of TRB3 cleavage in the apoptotic process to address the above question. Overexpression studies revealed that the cleavage of TRB3 promoted CASP3/7 activation and apoptosis. In contrast, the anti-apoptotic effects were found under TRB3 non-cleavable conditions, such as ER stress, and also when the CASP3/7 activation was enhanced by knockdown of endogenous TRB3 expression. Interestingly, nuclear translocation of procaspase-3 (proCASP3) was observed in cells either overexpressing TRB3 or under tunicamycin-induced ER stress. Although forced cytoplasmic expression of proCASP3 enhanced apoptosis significantly, its nuclear expression did not produce any pro-apoptotic effect, suggesting that nuclear distribution of proCASP3 is not critical for the execution of apoptosis. Thus, TRB3 might prevent cytoplasmic activation of CASP3 by promoting proCASP3 entry into the nucleus, and thereby inhibit apoptosis. Taken together, our results suggest that TRB3, through its own cleavage, functions as a molecular switch between the cell survival and apoptotic pathways under stressful conditions.

## Introduction

TRB3 (also known as TRIB3, NIPK, SINK, or SKIP), one of the mammalian orthologues of *Drosophila* Tribbles, was identified as a pseudokinase, because it contains a Ser/Thr protein kinase-like domain that lacked the ATP-binding domain and core catalytic residues, therefore, dose not have any kinase activity [1]. Despite a lack of characteristic functional domain, TRB3 has been shown to be involved in multiple cellular processes such as glucose and lipid metabolism, muscle and adipocyte differentiation, and stress response by interacting with various functional proteins (e.g. kinase: AKT, MAPK; transcription factor: ATF4, CHOP, PPARγ; E3 ubiquitin ligase: COP1) [2]–[9].

Endoplasmic reticulum (ER) stress has recently been recognized as another key pathway for triggering apoptosis [10], [11]. The adaptive phase of ER stress promotes cell survival by reducing the accumulation of unfolded proteins through global transcriptional control, well known as the unfolded protein response (UPR) [12]. However, apoptosis is considered selected when the apoptotic pathway gains ascendancy over the adaptive pathway by overwhelming the ER stress. During ER stress, TRB3 is upregulated by an ER stress-inducible transcription factor, ATF4/CHOP [6]. Excess expression of TRB3 downregulates its own expression by negative feedback via the repression of ATF4/CHOP transcriptional activity [13]. Several studies suggest that CHOP and its transcriptional target, BH3-only proteins such as Bim and PUMA, promote ER stress-induced apoptosis [14], [15]. TRB3 has been shown to be involved in ER stress-induced apoptosis via these regulatory processes [6], [16]. TRB3 expression is also induced in a PI3K-dependent manner by nutrient deficiency, such as the lack of glucose or amino acids [17]. Results of a transient overexpression study suggest that TRB3 plays an apoptosis inhibitory role under glucose depletion condition. Thus, the expression of TRB3 could be both up- and down-regulated by various cellular stresses [18]. Taken together, these studies indicate that TRB3 functions as an important component of the stress response mechanism, namely, regulates stress-induced apoptosis. However, it remains to be elucidated how TRB3 contributes to the stress responses.

Caspase-3 (CASP3), one of the most downstream components of the caspase cascade, is known to cleave many critical proteins such as lamin, PARP, ICAD/DFF45 and PAK2, and in turn induces irreversible apoptosis that involves substrate proteolysis and positive feedback of caspase cascade [19], [20]. Recently, we have demonstrated that TRB3 is a substrate for CASP3 [21]. To investigate the role of TRB3 cleavage in the apoptotic process, we carried out cell-based analysis using the wild type and a non-cleavable mutant of TRB3. In this study, we have shown a TRB3 cleavage-dependent pro-apoptotic response, and have also presented evidence for a novel anti-apoptotic mechanism involving TRB3-mediated nuclear translocation of procaspase-3 (proCASP3). This dual function of TRB3 may serve as a key switch between the cell survival and apoptosis pathways depending on the cellular context.

## Results

### TRB3 is Cleaved by Caspases in vitro and in the Apoptotic Process

**Figure 1:**
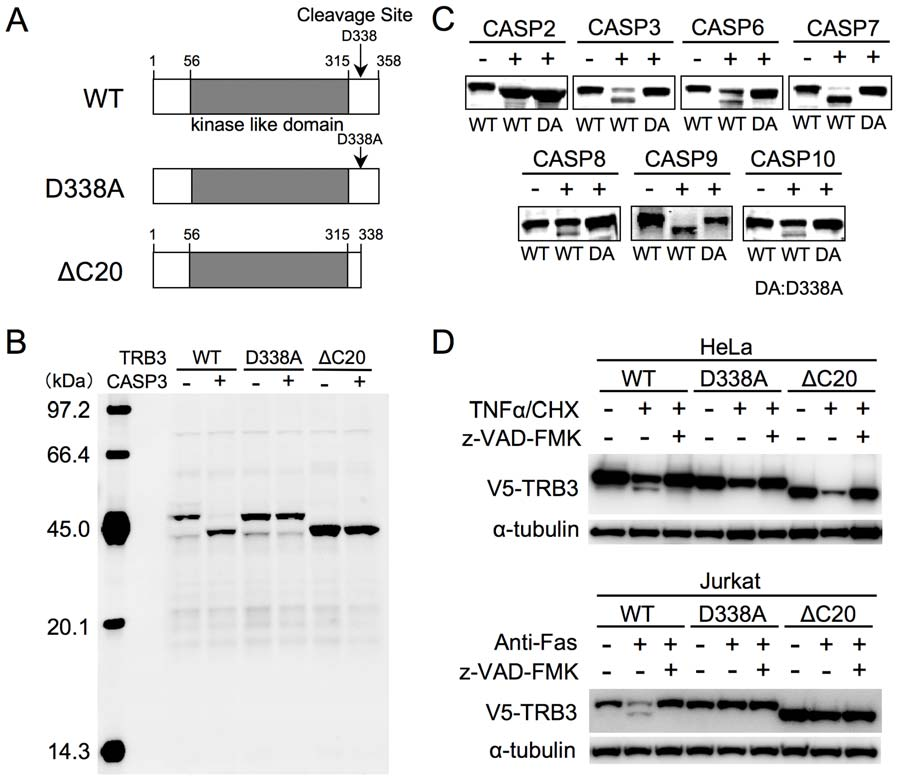
TRB3 is cleaved by caspases in vitro and in the apoptotic process. (A) Schematic representation of proteins expressed by the recombinant TRB3 constructs used in this study. WT, wild type TRB3; D338A, CASP3 cleavage-site mutant TRB3; and ΔC20, CASP3-cleaved form of TRB3. (B and C) Each recombinant TRB3 was synthesized and biotinylated using the wheat cell-free expression system as described in the Materials and Methods. The translation mixture was incubated with or without the indicated active caspase for 2 hr at 30°C. For the detection of N-terminal biotinylated-TRB3, the blot was probed with Alexa 488-conjugated streptavidin. DA denotes D338A-TRB3. (D) Twenty-four hours after transfection of HeLa and Jurkat cells with the indicated V5-tagged TRB3 expression plasmid, cells were treated with TNFα (20 ng/mL)/CHX (100 µM) (HeLa cells) or anti-Fas antibody (125 ng/mL) (Jurkat cells) in the absence or presence of z-VAD-FMK (100 µM) for 4 hr. DMSO was used as a treatment control. The cell lysates were subjected to immunoblot analysis using anti-V5 antibody to detect the N-terminal V5-tagged TRB3. α-Tubulin was used as an internal control.

We previously reported that TRB3 is cleaved by CASP3 at Asp338 [21]. To analyze the biological consequence of TRB3 cleavage by caspase, we constructed three recombinant plasmids for expressing the wild type (WT), CASP3 cleavage-site mutant (D338A) and CASP3-cleaved form (ΔC20) of TRB3, respectively, cartoon diagrams of which are shown in Figure 1A. As was shown in our previous report [21], the WT-TRB3 was cleaved by CASP3 *in vitro* (Figure 1B). In contrast, the D338A-TRB3 and ΔC20-TRB3 were not cleaved by CASP3. This result suggests that TRB3 was cleaved at a single site by CASP3. We also tested CASP2, CASP6, CASP7, CASP8, CASP9 and CASP10 for their ability to cleave TRB3 in this *in vitro* assay (Figure 1C). Surprisingly, all caspases used in this assay cleaved the WT-TRB3, and all of them, except for CASP2, were unable to cleave the D338A mutant. As judged from the mobility of the cleaved fragment on the SDS/PAGE (Figure 1C), the CASP2 cleavage site must be more close to the C-terminal end of TRB3 than the Asp338 residue, and we predicted that this site could be either at Asp343 and/or at Asp351. These results suggest that TRB3 is cleaved by multiple caspases, and the primary cleavage site is located at Asp338. Sequence alignment analysis of the TRB3 caspase-cleavage sites from multiple species raised a possibility that TRB3 is cleaved in various species and the cleavage has biological significance (see Figure S1 for details).

To examine whether TRB3 is cleaved in apoptosis-induced cells, we transfected HeLa and Jurkat cells separately with the recombinant TRB3 plasmids expressing WT-TRB3, D338A-TRB3, and ΔC20-TRB3. Cleavage of WT-TRB3 was observed in cells that were made apoptotic by treatment with tumor necrosis factor-α and cycloheximide (TNFα/CHX) (HeLa cells) or anti-Fas antibody (Jurkat cells), and this process was blocked by z-VAD-FMK, a caspase inhibitor (Figure 1D). As a result of *in vitro* experiment, the cleavage fragment derived from D338A-TRB3 and ΔC20-TRB3 was not detected in the apoptotic cells. Although the reduction of TRB3 levels was observed upon TNFα/CHX treatment, it may be due to proteasomal degradation and the rescue of protein levels seems to be the unexpected effect of z-VAD-FMK [21], [22]. However, it could not be denied that lack of ΔC20-TRB3 stability is caused by the deletion. Taken together, these results suggested that TRB3 was cleaved at Asp338 by caspases during apoptosis.

### Cleavage of TRB3 Promotes Apoptosis along with CASP3 Activation

**Figure 2:**
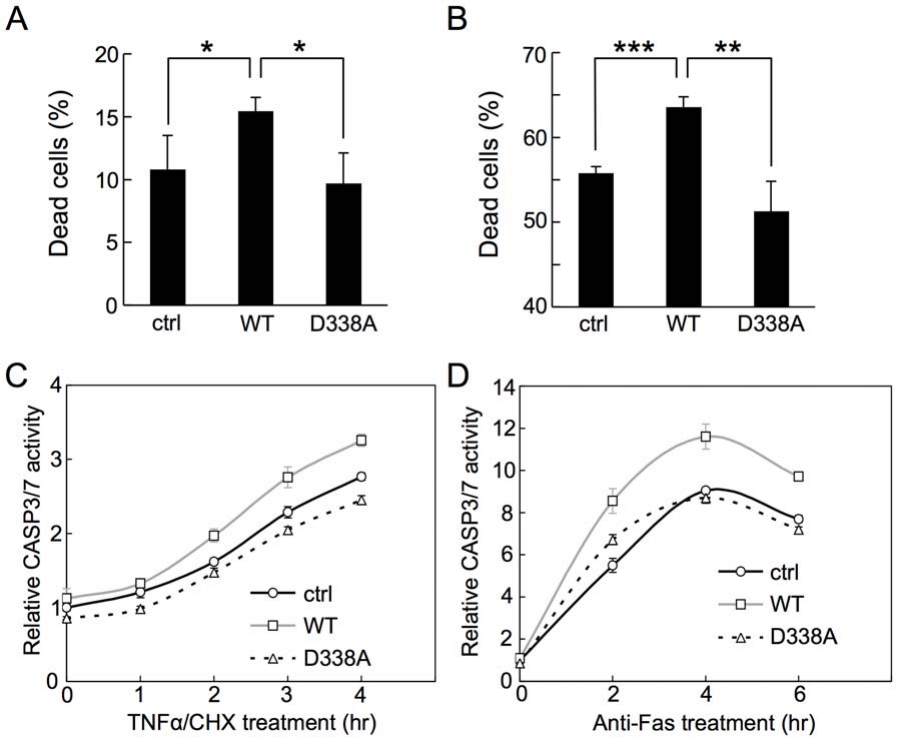
Cleavage of TRB3 promotes apoptosis along with CASP3 activation. (A and B) Twenty-four hours after transfection of HeLa (A) and Jurkat (B) cells with the indicated V5-TRB3 expression plasmid or control (ctrl) vector, the cells were treated with TNFα/CHX for 4 hr (HeLa cells) or anti-Fas antibody for 6 hr (Jurkat cells). The resulting dead cells were counted by trypan blue staining. Error bars indicate mean ±SD of three independent experiments. *P<0.05, **P<0.005, ***P<0.001, statistically significant difference. (C and D) Twenty-four hours after transfection with the indicated V5-TRB3 expression plasmid or control vector, HeLa (C) and Jurkat (D) cells were reseeded in 96-well plates (1.0×10^4^ cells/well), and then treated with TNFα/CHX and anti-Fas antibody, respectively, for the indicated times and CASP3/7 activity was then measured using the luminometric Caspase-Glo® 3/7 Assay Kit. Each data point represents mean ±SD of three independent experiments.

TRB3 has been shown to be involved in the regulation of apoptosis under stress conditions. For example, overexpression of TRB3 under ER stress conditions produced pro-apoptotic effect [6]. Exact contribution of TRB3 to apoptosis, however, remains unclear at present. To determine how TRB3 cleavage affects apoptosis, we transiently overexpressed the cleavable TRB3 (WT) and non-cleavable TRB3 (D338A) in HeLa and Jurkat cells. Accordingly, both cells were separately transfected with the recombinant plasmids expressing WT-TRB3 or D338A-TRB3, or with the pcDNA3.1 empty vector (control vector), transfected cells were then treated with TNFα/CHX (HeLa cells) or anti-Fas antibody (Jurkat cells) to induce apoptosis and subsequently dead cells were counted. As shown in Figure 1D, expression levels of WT-TRB3 and D338A-TRB3 in both cells were very similar. Interestingly, the percentage of dead cells was higher in the WT-TRB3 expressing cells, but not in the D338A-TRB3 expressing cells, as compared to that in the control cells (Figure 2A and B). In both cells, the reagent-induced cell death was rescued back to the untreated control level when the stimuli were performed in the presence of the caspase inhibitor z-VAD-FMK (Figure S2), suggesting that cell death was due to apoptosis. These results suggest that the pro-apoptotic effect of TRB3 depends on its cleavage by caspases.

It is possible that the cleavage of TRB3 is an important event in the pro-apoptotic process. Because CASP3 activation is a critical step in the execution of apoptosis, we next investigated the effect of TRB3 cleavage on CASP3 activation. We used a luminescence-based assay to measure the CASP3/7 activity in cells. As shown, the CASP3/7 activity was found to be higher in the WT-TRB3 expressing cells, but not in the D338A-TRB3 expressing cells, than that in the control cells (Figure 2C and D). Taken together, these results suggest that cleavage of TRB3 is required for inducing further activation of CASP3 and/or CASP7, and in turn might promote apoptosis.

### TRB3 Inhibits CASP3 Activation and Subsequent Apoptosis under ER Stress Condition

**Figure 3:**
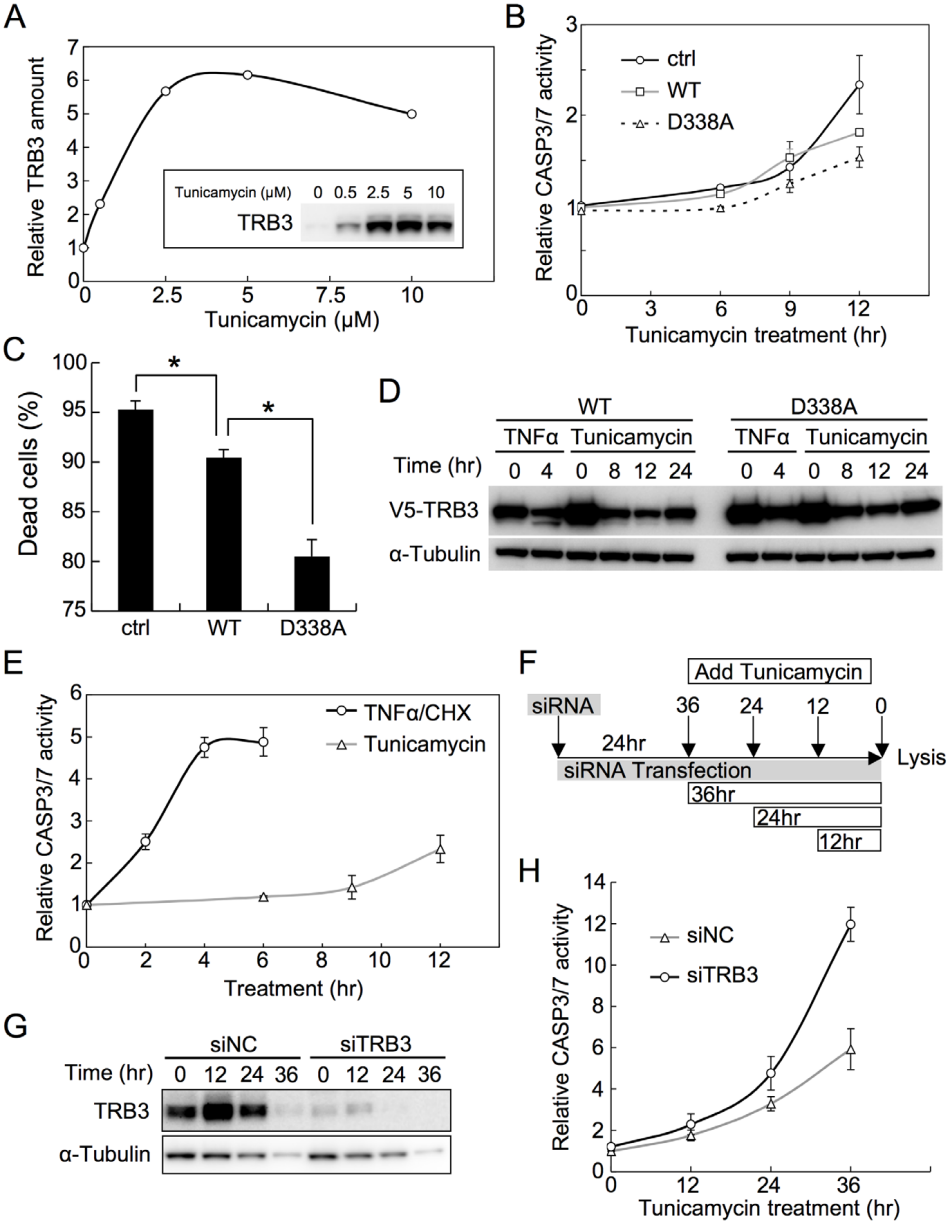
TRB3 inhibits CASP3 activation and subsequent apoptosis under ER stress condition. (A) HeLa cells were treated with the indicated concentrations of tunicamycin for 8 hr. The effective concentration of tunicamycin needed for inducing TRB3 expression was determined by densitometric image analysis. (B) CASP3/7 activity in HeLa cells was measured as described in the legend of Figure 2C, except that here apoptosis was induced by incubation with 5 µM tunicamycin for the indicated times. Each data point represents mean ±SD of three independent experiments. (C) Twenty-four hours after transfection with the indicated V5-TRB3 expression plasmid or control vector, HeLa cells were treated with tunicamycin for 48 hr. The resulting dead cells were counted by trypan blue staining. Error bars indicate mean ±SD of four independent experiments. *P<0.001. (D) Twenty-four hours after transfection with the given V5-TRB3 expression plasmid, HeLa cells were treated with TNFα/CHX or tunicamycin for the indicated times. The cell lysates were subjected to immunoblot analysis using the anti-V5 antibody. α-Tubulin was used as an internal control. (E) HeLa cells plated in 96-well plates (1.0×10^4^ cells/well) were treated with TNFα/CHX or tunicamycin for the indicated times. CASP3/7 activity was measured using the luminometric Caspase-Glo® 3/7 Assay Kit. Each data point represents mean ±SD of three independent experiments. (F-H) HeLa cells were transfected with negative control siRNA (siNC) or TRB3 siRNA (siTRB3). Twenty-four hours after, the cells were treated with tunicamycin for the indicated time points (F). The cell lysates were subjected to immunoblot analysis using the anti-TRB3 antibody (G). Cell lysates were also used for the Caspase-Glo® 3/7 assay to measure the CASP3/7 activity (H). The relative CASP3/7 activity was then determined after normalizing each value with respect to the relative amount of expressed α-Tubulin as estimated by densitometric image analysis. Each data point represents mean ±SD of three independent experiments.

Tunicamycin treatment, which causes ER stress in cells, has been shown to induce TRB3 expression in several cell lines [6], [16]. TRB3 expression was indeed induced in HeLa cells treated with 5 µM tunicamycin for 8 hours (Figure 3A), and the disappearance of endogenous TRB3 was clearly observed in apoptotic cells, although cleaved fragment was not observed due to use of C-terminus recognition antibody. Other antibodies could not also detect the cleaved fragment (Figure S3). Therefore, we next used tunicamycin treatment to examine how TRB3 cleavage affects CASP3/7 activation and subsequent apoptosis under ER stress conditions. D338A-TRB3 expressing cells were resistant to CASP3/7 activation and death (Figure 3B and C). Surprisingly, we also observed anti-apoptotic effect, rather than pro-apoptotic effect, in WT-TRB3 expressing cells, although this anti-apoptotic effect was lower than that in D338A-TRB3 expressing cells (Figure 3B and C). As previously reported [6], a major portion of the tunicamycin-induced cell death also appears to be apoptotic cell death, because cell death was strongly inhibited in the presence of z-VAD-FMK (Figure S4). Unlike in the case of TNFα/CHX- or anti-Fas antibody-induced apoptosis (as shown in Figure 2), WT-TRB3 actually rescued cells from death under ER stress condition. To explain this result, we examined the cleavage of WT-TRB3 during ER stress. We found hardly any cleavage of WT-TRB3 in tunicamycin-treated cells (Figure 3D), although it was clearly seen in TNFα/CHX- and anti-Fas antibody-treated cells (Figure 1D and 3D). Most of the subsequent reduction of WT-TRB3 after tunicamycin treatment may be not resulting consequence by cleavage because TRB3 is rapidly degraded through the ubiquitin-proteasome pathway as previously described [22] and non-cleavable TRB3 (D338A) was also reduced in a similar manner. Moreover, ER stress regulates protein synthesis via the unfolded protein response (UPR). Therefore, the global reduction of protein levels could be caused by ER stress condition itself. Next, to clarify the observed difference in susceptibility to cleavage, we measured the CASP3/7 activity in TNFα/CHX- or tunicamycin-treated cells. The increase in CASP3/7 activity was very slow during the tunicamycin-induced ER stress, and was significantly lower than in cells undergoing apoptosis induced by TNFα/CHX treatment (Figure 3E). This result suggests that the ER stress-induced CASP3/7 activity, at least in this condition, is not so high as to actively cleave TRB3.

To assess whether TRB3 itself is required for the modulation of CASP3/7 activation, we used siRNA to knockdown the TRB3 expression in cells under ER stress condition (Figure 3F). As shown, TRB3 siRNA (siTRB3) specifically reduced the expression of endogenous TRB3 upto 24 hours following the tunicamycin treatment (Figure 3G), by which time the relative activity of CASP3/7 increased over the activity found in negative control siRNA (siNC)-transfected cells (Figure 3H). Taken together, these results strongly suggest that TRB3 intrinsically blocks CASP3 and/or CASP7 activation and subsequent apoptosis under TRB3 non-cleavable conditions, such as ER stress.

### TRB3 Expression Induces Nuclear Translocation of proCASP3

**Figure 4:**
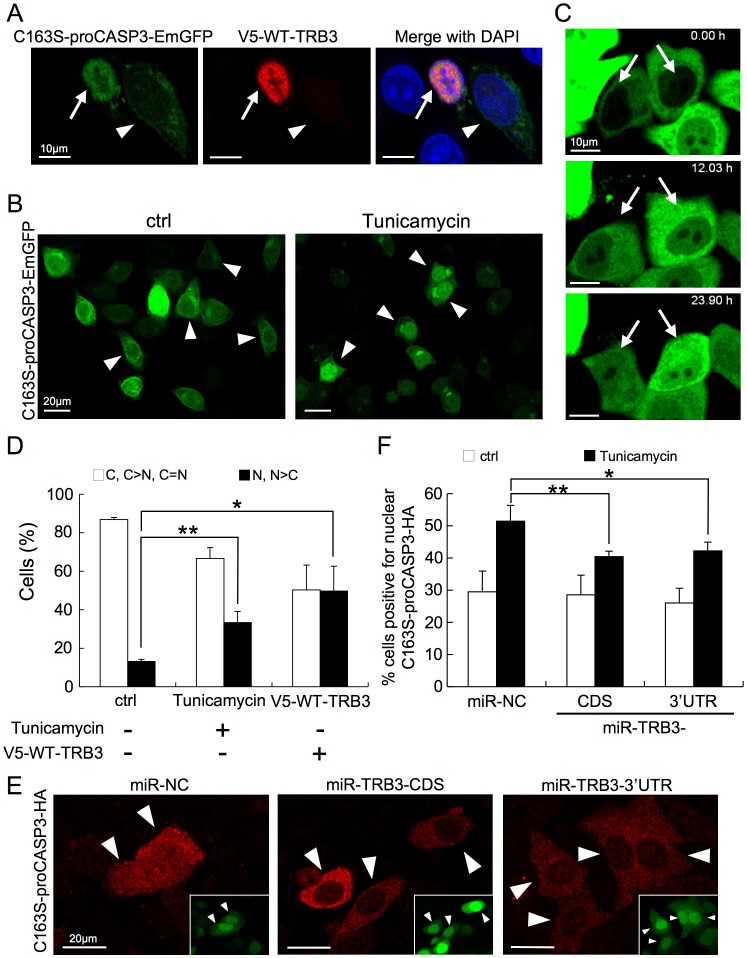
TRB3 expression induces nuclear translocation of proCASP3. (A) HeLa cells were cotransfected with the C163S-proCASP3-EmGFP and V5-WT-TRB3 expression plasmids. After 24 hr, the cells were stained with anti-V5 antibody (red) and counterstained with DAPI (blue). Arrow and arrowhead indicated V5-WT-TRB3 positive and negative cells respectively. Scale bars = 10 µm. (B) Twenty-four hours after transfection with the C163S-proCASP3-EmGFP expression plasmid, HeLa cells were incubated with or without tunicamycin for 8 hr. Arrowheads showed representative phenotypes of C163S-proCASP3-EmGFP localization in each condition. Scale bars = 20 µm. (C) Twenty-four hours after transfection with the C163S-proCASP3-EmGFP expression plasmid, HeLa cells were treated with tunicamycin, and localization of C163S-proCASP3-EmGFP was simultaneously monitored for 24 hr by live imaging. Representative frames displaying scenes of nuclear translocation (arrows) are shown. Scale bars = 10 µm. (D) Localization of C163S-proCASP3-EmGFP was quantified as cytoplasmic, mainly cytoplasmic or cytoplasmic equal to nuclear (C, C>N, C = N), or as nuclear or mainly nuclear (N, N>C) under conditions described above (Figure 4A, V5-WT-TRB3; Figure 4B, ctrl or Tunicamycin). This localization was also assessed in cells coexpressing V5-WT-TRB3 (right bar graph). Over 30 cells were assessed in three independent experiments. Error bar: mean ±SD. *P<0.01, **P<0.005. (E) HeLa cells were cotransfected with the C163S-proCASP3-HA and indicated artificial miRNA expression plasmids. After 24 hr, the cells were incubated with tunicamycin for 8 hr. The localization of C163S-proCASP3-HA was observed by immunofluorescence staining with an anti-HA antibody (red). The miRNA expressing cells of inner panel were visualized by cocistronic expression of EmGFP. Arrowheads showed the C163S-proCASP3-HA expressing cells. Scale bars = 20 µm. (F) The cells positive for nuclear C163S-proCASP3-HA (N, N>C, N = C) were quantified by immunofluorescence staining with an anti-HA antibody. Over 30 cells positive for EmGFP were assessed in six independent experiments. Error bar: mean ±SD. *P<0.005, **P<0.001. miR-NC denotes negative control miRNA.

In Figure 3, we have presented evidence that suggests that TRB3 negatively regulates the activation of CASP3. However, the premature CASP3 (i.e. procaspase-3; proCASP3) and TRB3 are differently localized in cells; proCASP3 is predominantly localized in the cytoplasm [23], [24], whereas TRB3 is localized in the nucleus [5], [7]. Thus, even though TRB3 is a substrate for CASP3, how these two molecules would interact with each other still remains elusive. To gain further insights about that, we next examined whether the subcellular localization of proCASP3 would be affected by TRB3 expression. For this purpose, we constructed a recombinant plasmid expressing an EmGFP-tagged inactive proCASP3 mutant (C163S-proCASP3-EmGFP), in which the active site cysteine residue was altered to a serine residue (C163S) to prevent apoptosis. We next cotransfected HeLa cells with the C163S-proCASP3-EmGFP and WT-TRB3 expression plasmids, and subsequently assessed the subcellular localization based on the relative fluorescence intensity of EmGFP by using confocal microscopy. Under normal culture condition, C163S-proCASP3-EmGFP predominantly exhibited cytoplasmic localization (left panels in Figure 4A and B, see arrowheads), which is consistent with the known localization of proCASP3. Interestingly, C163S-proCASP3-EmGFP was found mainly in the nucleus of cells coexpressing WT-TRB3 (Figure 4A, see arrow).

As shown in Figure 3A, under normal culture condition cells maintained the endogenous TRB3 expression at a relatively low level, which significantly increased after tunicamycin treatment for 8 hours. So next, we examined the localization of C163S-proCASP3-EmGFP under tunicamycin-induced ER stress condition. Because active CASP3 was hardly detected by immunofluorescence in tunicamycin-treated morphologically normal cells (98.7% were active CASP3 negative; 100 cells were assessed in three independent experiments), we assumed that C163S-proCASP3-EmGFP as the premature form (i.e. proCASP3) in these cells (Figure S5). Increased amount of C163S-proCASP3-EmGFP was found in the nucleus of tunicamycin-treated cells (Figure 4B right panel, see arrowheads). Furthermore, we could monitor the nuclear accumulation of C163S-proCASP3-EmGFP by live imaging of cells up to 24 hours following the tunicamycin treatment (Figure 4C). The number of cells expressing nuclear C163S-proCASP3-EmGFP was increased by tunicamycin treatment and also by coexpression of WT-TRB3 (Figure 4D). The difference in the cytoplasmic/nuclear ratio of C163S-proCASP3-EmGFP between the tunicamycin-treated and WT-TRB3-overexpressed cells suggests that the nuclear translocation efficiency of C163S-proCASP3-EmGFP might be dependent on the TRB3 expression level. A similar phenotype was observed in D338A-TRB3 expressing cells (Figure S6).

Using cells expressing HA-tagged inactive proCASP3 mutant (C163S-proCASP3-HA), we also obtained similar results of the localization before and after tunicamycin treatment (Figure S7). Therefore, we further examined whether the efficiency of nuclear translocation of C163S-proCASP3-HA would decrease by microRNA (miRNA)-mediated down-regulation of endogenous TRB3 expression. To this end, we designed two types of artificial miRNA targeting TRB3 mRNA (miR-TRB3-CDS and miR-TRB3-3′UTR), and which specifically suppressed endogenous TRB3 expression (Figure S8). We could identify the miRNA expressing cells by cocistronic expression of EmGFP from the same plasmid. The localization of C163S-proCASP3-HA was assessed in EmGFP expressing cells (Figure 4E and F). Consequently, the phenotype of cells positive for nuclear C163S-proCASP3-HA was decreased in miR-TRB3 expressing cells under tunicamycin-induced ER stress condition. Taken together, these results suggest that ER stress induced TRB3 expression leads to nuclear translocation of proCASP3.

### Nuclear proCASP3 did not Promote Apoptosis

**Figure 5:**
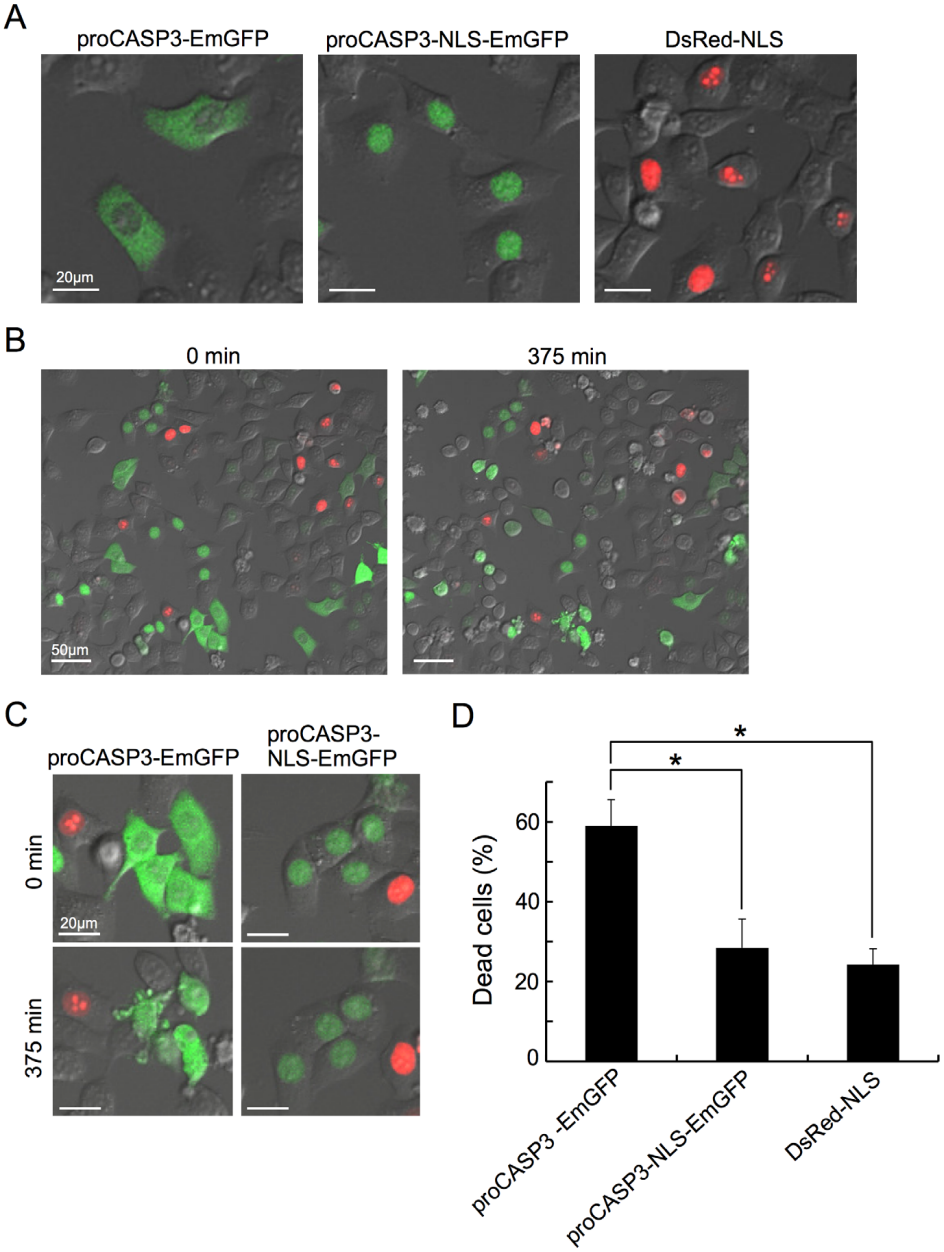
Nuclear proCASP3 did not promote apoptosis. (A–D) HeLa cells were separately transfected with the plasmid expressing EmGFP-tagged wild type proCASP3 (proCASP3-EmGFP), nuclear localization signal (NLS) containing proCASP3-EmGFP (proCASP3-NLS-EmGFP), or DsRed-NLS fusion protein (DsRed-NLS). After 24 hr, transfected cells were trypsinized and plated together in one glass bottom dish. After a further 24 hr, apoptosis was induced by the addition of anti-Fas antibody. Cells expressing the indicated fusion protein are shown (A). Behavior of the EmGFP-fusion protein positive and DsRed positive cells (same field at different times) was recorded by live imaging (B). Representative frames showing images of cells expressing the indicated fusion protein in a given field, as recorded in Figure 5B, are displayed (C). Scale bars = 20 µm (A and C), 50 µm (B). The number of apoptotic cells (based on cell morphology) was counted at 450 min after apoptosis induction by anti-Fas antibody (D). A minimum number of 30 cells were counted in a field for each type of fusion protein expressing cells. Four fields were randomly chosen for counting the number of apoptotic cells. Error bar: mean ±SD. *P<0.001.

Results shown in Figure 3 and 4 suggested that TRB3 expression produced anti-apoptotic effect and it also induced nuclear translocation of proCASP3 under ER stress condition. We hypothesized that inhibition of apoptosis by TRB3 resulted from the nuclear translocation of proCASP3. Paradoxically speaking, the presence of proCASP3 in the cytoplasm, but not that in the nucleus, appears to be more important for the execution of apoptosis, because it has been proposed that the activation of CASP3 initially occurs in the cytoplasm, and the activated CASP3 then gets translocated to the nucleus [25]. To validate our hypothesis, we constructed three recombinant plasmids – one expressing EmGFP-tagged wild type proCASP3 (proCASP3-EmGFP), the second expressing nuclear localization signal (NLS) containing proCASP3-EmGFP (proCASP3-NLS-EmGFP) and the third expressing DsRed-NLS fusion protein (DsRed-NLS; control), and used them to investigate the effect of forced nuclear expression of proCASP3 on apoptosis. Accordingly, HeLa cells were separately transfected with these plasmids, and then the transfected cells were plated together in one glass bottom dish. After inducing apoptosis, EmGFP-fusion protein positive and DsRed positive cells were observed by live imaging. As expected, under normal culture condition (i.e. cells not fixed), proCASP3-EmGFP was localized in the cytoplasm (Figure 5A left panel), whereas proCASP3-NLS-EmGFP (middle panel) and DsRed-NLS (right panel) were localized only in the cell nucleus. The anti-Fas antibody was used for apoptosis induction, because it did not induce nuclear translocation of proCASP3 at least during imaging. As shown in Figure 5B and C, apoptotic cell death, based on cell morphology, was preferentially induced in cells expressing the cytoplasmic proCASP3 (proCASP3-EmGFP) than in cells expressing the nuclear proCASP3 (proCASP3-NLS-EmGFP) or the nuclear DsRed-NLS (Figure 5D and see Movie S1). These results strongly suggest that nuclear distribution of proCASP3 is not critical for the execution of apoptosis. Taken together with the previous section, our results suggest that TRB3 expression prevents CASP3 activation by nuclear translocation of proCASP3, and thereby inhibits apoptosis.

## Discussion

TRB3 expression is up-regulated in a variety of cell types under various stress conditions including ER stress, nutrient deprivation, hypoxia and oxidative stress [1], [6], [17], [26]. However, the exact role of TRB3 expression in stress response is controversial because it has been reported that the stress-induced expression of TRB3 exhibited both pro-apoptotic [6], [16], [27], [28] and anti-apoptotic [17], [26] effects. Several studies have led to the suggestion that the opposite behavior of TRB3 was due to its putative role as a stress sensor [6], [13], [29]. The exact molecular mechanism, however, remains unclear.

Recently, we found that TRB3 was cleaved by CASP3, and this observation could easily be correlated to the previous reports by suggesting that TRB3 is involved in the regulation of apoptosis. In this study, we further found that the C-terminal region of TRB3 was a target for most caspases (Figure 1C). In fact, the expressed TRB3 was cleaved within a few hours of inducing apoptosis using TNFα/CHX or anti-Fas antibody (Figure 1D and 3D). In this case, CASP3/7 activation and subsequent apoptosis were promoted in WT-TRB3 expressing cells, but the pro-apoptotic effect of TRB3 was not observed in cells expressing the non-cleavable D338A-TRB3 (Figure 2). Additionally, we have found that ΔC20-TRB3 (see Figure 1A), which could be produced by multiple caspases during the apoptotic process (Figure 1C and D), did not have any pro-apoptotic effect (Figure S9), suggesting that the pro-apoptotic effect is not mediated by the cleaved TRB3 (ΔC20) but likely requires the C-terminal region of TRB3 and/or the cleavage event to function. Indeed, a peptide, around 10 residues long, such as amyloid β-peptide, has been shown to induce apoptosis [30], [31]. In contrast to the above case, TRB3 cleavage was hardly observed even if the cells were exposed to ER stress by tunicamycin for as long as 24 hours (Figure 3D). CASP2 has been shown to be involved in ER stress with treatment of high tunicamycin condition (>20 µg/mL = 24 µM) [32], [33] and has TRB3 cleavage ability (Figure 1C). However CASP2 activation may be not found in low tunicamycin condition (<5 µg/mL = 6 µM) [32]. Actually, in our condition (5 µM) it was not detected (data not shown), and TRB3 cleavage was also hardly observed (Figure S10A), like Figure 3D. In addition, treatment of CASP2 inhibitor (z-VDVAD-FMK) rescued only small number of the dead cells (Figure S10B). Therefore, at least in our condition, CASP2 seems to have only limited impact on apoptosis by tunicamycin. Interestingly, in these cases, CASP3/7 activation and subsequent apoptosis were slightly inhibited in WT-TRB3 expressing cells (Figure 3B, C and S10B), and the results shown in Figure 4 and 5 suggested that the anti-apoptotic effect was due to the TRB3-mediated nuclear translocation of proCASP3. The non-cleavable D338A-TRB3 was also localized in the nucleus (Figure S6A), and its translocation efficiency of proCASP3 was almost same as that by WT-TRB3 (Figure S6B). But, in the case of chronic stress, the anti-apoptotic effect of WT-TRB3 was lower than that of D338A-TRB3 (Figure 3B and C), likely as a result of the combined pro-apoptotic effect of the cleavable TRB3 (i.e. WT-TRB3). To investigate how TRB3 affects apoptosis induced by intrinsic stimulus independent of ER stress, we further used staurosporine (STS), a wide spectrum inhibitor of protein kinase. As well as the case of ER stress condition, TRB3 exerted the anti-apoptotic effect on STS-treated cells at least in our condition (Figure S11A), and in which TRB3 cleavage was also hardly observed (Figure S11B and C), even though STS-induced apoptosis is essentially mediated by CASP9 [34] that has TRB3 cleavage ability (Figure 1C). These results support our hypothesis that the anti-apoptotic effect of TRB3 is exerted during adaptive phase of stress as long as TRB3 is not cleaved. Taken together, our findings indicate that TRB3 has dual function – both in cell survival and also in apoptosis. This dual function of TRB3 might have contributed to the previously reported controversial role of TRB3 in apoptosis.

Initiator caspases, especially CASP8, CASP9, and CASP10, are known to play important roles in the beginning of apoptotic signal transduction [35]. Activated initiator caspases could then lead to the activation of downstream, effector caspases (i.e. CASP3, CASP6, and CASP7). Most caspases, including CASP8, CASP9, and CASP10 are predominantly located in the cytoplasm, and not in the nucleus [36], [37]. Using fluorescence resonance energy transfer (FRET) technique it was shown that during the apoptotic process CASP3 activation is first initiated in the cytoplasm and then in the nucleus [25]. These results suggest that the cytoplasmic initiator caspases cannot easily activate the nuclear proCASP3. In fact, our results showed that expression of the nuclear proCASP3 (proCASP3-NLS-EmGFP) did not enhance cell death under apoptotic conditions compared with control (DsRed-NLS), although expression of the cytoplasmic proCASP3 (proCASP3-EmGFP) enhanced it markedly (Figure 5). We found that TRB3 expression can induce cytoplasmic-to-nuclear translocation of proCASP3 under ER stress condition (Figure 4). TRB3 is a stress-inducible protein, and stress response is a mechanism by which cells adapt to stressful situations. Thus, TRB3 might insulate proCASP3 by promoting its entry into the nucleus to prevent CASP3 activation in the cytoplasm under TRB3-inducing stress conditions. Taken together, these results suggest that the overall CASP3 activity is regulated not only by proteolytic activation of proCASP3 but also by its subcellular distribution. To the best of our knowledge, TRB3-mediated nuclear translocation of proCASP3 is the first evidence supporting the above idea. However, we could not confirm the direct interaction between TRB3 and proCASP3 either by *in vitro* binding assay or by immunoprecipitation using the overexpressing cells. Further studies are required for better understanding of the mechanism.

Our results indicated that TRB3 behaved as both an anti-apoptotic and a pro-apoptotic factor by whether or not TRB3 is cleaved by caspases under stressful situations. We propose the following model for the stress response mechanism of TRB3 (Figure S12). During the adaptive phase of stress, expressed TRB3 prevents CASP3 activation by nuclear translocation of proCASP3, which in turn leads to an extended adaptive phase and cell survival (Figure 3 and 4). However, when the stress becomes overwhelming, such as under prolonged stress, TRB3 is cleaved by caspases. According to indirect evidence, cleavage of TRB3 could be a trigger for further activation of caspases and thereby induces apoptosis (Figure 2), even if the condition that a part of proCASP3 had been localized to the nucleus by TRB3 (Figure 4A). Therefore, it seems like the anti-apoptotic effect of TRB3 had been abrogated when caspase activity is beyond tolerance level by lethal apoptotic stimuli such as TNFα/CHX unlike tunicamycin.

During ER stress, “because the UPR controls cell fate by switching between pro-survival and pro-apoptotic signaling, it is crucial to understand the parameters or events defining this transition, as well as gain insights on the components involved in its regulation” [12]. The exact mechanism controlling the transition between the adaptive and cell death programs is not well understood. In this study, we propose that TRB3 functions as a stress sensor that detects caspase activity and also as a switch molecule that assists cells to commit to either survival or apoptotic pathway, and these functions of TRB3 are exerted through its own cleavage by caspases depending on the cellular context. Thus, TRB3 may play an important role in this strategic commitment mechanism.

Recently, it has been reported that CASP3 have cellular functions other than apoptosis, such as in cell development and differentiation [35]. In fact, during the development of iPS cells, CASP3/8 were activated and cleaved retinoblastoma (Rb), a known transcription coactivator [38]. The cleavage of Rb by CASP3/8 enhanced iPS formation. Among the initiator caspases, proCASP2 has been identified in the cell nucleus [39], [40]. This raises a possibility that proCASP3 is actually activated in the nucleus via the mediation of TRB3, and that transcriptional regulators, such as Rb, could be targets of the nuclear form of active CASP3 under nonlethal condition (i.e. TRB3 non-cleavable conditions for nonapoptotic function). Alternatively, apoptosis accompanied by iPS formation is also suggestive of pro-apoptotic effect of TRB3. In another report, designed ER stress enhanced differentiation-associated apoptosis of myoblasts [41]. It is likely that ER stress-induced apoptosis selectively eliminates vulnerable cells. Interestingly, surviving myoblasts were resistant to apoptosis and they differentiated well. This result could be related to our finding that ER stress-induced TRB3 expression exerted anti-apoptotic effect by translocating proCASP3 into the nucleus. Because TRB3 expression is induced by various types of stresses, these results suggest that proCASP3 localizes in the nucleus under a variety of stress conditions. That proCASP3 was found to be localized in the nucleus, could now open avenues for finding new insights on biological roles of CASP3.

## Materials and Methods

### Plasmid Constructions

Full-length human TRB3 cDNA and cDNA coding for amino acids 1–338 of human TRB3 (ΔC20-TRB3) were amplified by PCR. To construct the expression plasmids pcDNA3.1-V5-WT-TRB3 and pcDNA3.1-V5-ΔC20-TRB3, the above cDNAs were individually subcloned into the pcDNA3.1nV5-DEST vector (Invitrogen, Carlsbad, CA, USA) via the donor vector pDONR221 of the Gateway Cloning Technology kit (Invitrogen). An expression plasmid containing a cDNA of a caspase non-cleavable D338A mutant of TRB3 (pcDNA3.1-V5-D338A-TRB3) was generated from the pcDNA3.1-V5-WT-TRB3 plasmid by using the PrimeSTAR Mutagenesis Basal kit (TakaraBio, Otsu, Japan) and utilizing the mutagenic primers (5′-GTCCCTGCGGGACTGGGGCTGGACGAA-3′ and 5′-CAGTCCCGCAGGGACCACCTGGGCAGC-3′) as described in the manufacturer’s instructions. In the same way, cDNAs of human caspase-3 and C163S mutant of caspase-3 (kindly provided by Dr. K. Sakamaki, Kyoto Univ.) were subcloned into the pcDNA6.2/C-EmGFP-DEST vector (Invitrogen) to construct the expression plasmids pcDNA6.2-proCASP3-EmGFP and pcDNA6.2-C163S-proCASP3-EmGFP, respectively. Three copies of the SV40 T-antigen nuclear localization signal (NLS) and HA tag sequences were fused by PCR to construct pcDNA6.2-proCASP3-NLS-EmGFP and pcDNA6.2-C163S-proCASP3-HA plasmids, respectively. The DsRed-NLS expression plasmid (pDsRed2-Nuc) was purchased from Clontech (Palo Alto, CA, USA).

### In vitro Cleavage Assay

Construction of DNA templates and cell-free protein synthesis for N-terminal biotinylated-TRB3 were performed as described previously [21]. For the cleavage reaction, 3 µl of translation mixture was added to 7 µl of reaction mixture (20 mM HEPES, pH 7.8, 100 mM NaCl, 10 mM DTT, 1 mM EDTA, 10% sucrose), with 0.3 (CASP3, CASP7, CASP8), 10 (CASP9) or 20 (CASP2, CASP6, CASP10) units of each active caspase (1 unit is defined as the amount that will hydrolyze 1 nmole of the caspase substrate per minute or hour) (Sigma-Aldrich, St. Louis, MO), and then the mixture was incubated for 2 hr at 30°C. The assay using CASP9 was performed in the reaction mixture containing 10% PEG6000 because it is necessary for the CASP9 activity. Additionally, to remove PEG6000 from the reaction mixture after cleavage reaction, the biotinylated-TRB3 was recovered by streptavidin magnetic beads (Promega Corporation, Madison, WI, USA). These reaction mixture and recovered-TRB3 were boiled in SDS-PAGE sample buffer. The samples separated on SDS-PAGE were transferred to PVDF membrane (Millipore, Bedford, MA, USA). The membrane was probed with Alexa 488-conjugated streptavidin (Invitrogen), and then visualized using a Typhoon Imager (GE Healthcare, Piscataway, NJ).

### Cell Culture and Transfection

HeLa and Jurkat cells were grown in Dulbecco’s modified eagle medium (DMEM) and RPMI medium, respectively. Each medium was supplemented with 10% fetal bovine serum (FBS), 100 units/mL penicillin and 100 µg/mL streptomycin. Transient transfection of HeLa and Jurkat cells with plasmid was carried out using Lipofectamine 2000 (Invitrogen) and FuGENE6 (Roche, Indianapolis, IN, USA), respectively, and following the manufacturer’s instructions. Empty vector pcDNA3.1 was used as a transfection control.

### Apoptosis Induction and Assay

To induce apoptosis, cells were treated with TNFα/CHX [20 ng/mL TNFα (Calbiochem, La Jolla, CA), 100 µM CHX (Chemicon, Temecula, CA)], 125 ng/mL anti-Fas antibody (IgM, CH11) (Medical & Biological Laboratories Co., Ltd., Nagoya, Japan) or 5 µM tunicamycin (Sigma-Aldrich) for various times as indicated in the figure legends. For inhibition of apoptosis, the above reagents were supplemented with 100 µM z-VAD-FMK (Peptide Institute Inc., Osaka, Japan). DMSO was used as a treatment control. The treated cells were subjected to each assay or suspended in an equal volume of 0.5% trypan blue solution (Nacalai Tesque, Kyoto, Japan) for 1 min at roomtemperature, and then the stained cells were counted as dead cells.

### Immunoblot Analysis

After washing with PBS, cells were lysed in 2× SDS-PAGE sample buffer (125 mM Tris-HC1, pH 6.8, 20% glycerol, 4% SDS, 10% 2-mercaptoethanol, 0.001% bromophenol blue) and the proteins in the cell lysates were heat denatured. Proteins in the cell lysates were separated by SDS-PAGE and then transferred to PVDF membrane (Millipore) by electroblotting. Membranes were subsequently used in immunoblot assay using one of the following primary antibodies: anti-V5 epitope (#R960-25, Invitrogen), anti-TRB3 (#2488-1, Epitomics, Burlingame, CA), and anti-α-tubulin (#T9026, Sigma-Aldrich). Chemiluminescent signals generated by Immobilon Western HRP substrate Luminol Reagent (Millipore) or ImmunoStar (Wako, Osaka, Japan), were detected using an LAS-4000 mini biomolecular imager (GE Healthcare).

### Densitometric Image Analysis

To measure the relative expression level of proteins in cells, acquired images were densitometrically analyzed by using the ImageJ software (NIH, Bethesda, MD, USA).

### Luminometric CASP3/7 Activity Assay

CASP3/7 activity was measured by using the luminometric Caspase-Glo® 3/7 Assay kit (Promega) and a GloMax™ 96 Microplate Luminometer (Promega) according to the manufacturer’s instructions. CASP3/7 activity shown was relative to the untreated control value.

### Knockdown of Endogenous TRB3 by Small Interfering RNA and microRNA

Negative control siRNA (5′-AATTCTCCGAACGTGTCACGT-3′) and siRNA oligonucleotide targeting 3′UTR of human TRB3 (5′-ATGAGGCTAGTTCTTGTCTAA-3′) were purchased from QIAGEN (Valencia, CA, USA). Transfection of cells with siRNA was performed according to the manufacturer’s instructions using TransIT-siQUEST transfection reagent (Mirus Bio Corporation, Madison, WI). For knockdown of human TRB3 using a miRNA interference, DNA duplexes targeting the TRB3 coding sequence (CDS) (5′-TTGGAGTTGGATGACAACTTA-3′) and 3′UTR (5′-CAGTTCCTGCTTGGGTGCTTA-3′) were designed by using Invitrogen BLOCK-iT RNAi Designer (at www.invitrogen.com/rnaidesigner) and cloned into pcDNA6.2-GW/EmGFP-miR expression vector (Invitrogen) that enable identify artificial miRNA expressing cells by cocistronic expression of EmGFP. Negative control miRNA expression vector was purchased from Invitrogen.

### Immunofluorescence Analysis

HeLa cells grown on coverslips were washed with cold PBS, and then fixed with 2% paraformaldehyde in PBS for 10 min. After washing with PBS, the fixed cells were permeabilized with 0.5% Triton X-100 (Nacalai Tesque) in PBS for 5 min. After washing with PBS, the cells were incubated in a blocking buffer [TBS containing 5% calf serum (Invitrogen)] for 1 hr. The cells were then incubated with one of the primary antibodies (listed below) in TBST containing 0.05% BSA for 1 hr at 37°C, washed three times with TBST (5 min each), and incubated with the Alexa Fluor 488/555-conjugated secondary antibody (Invitrogen) plus DAPI (Invitrogen) for 1 hr. After washing three times with TBST (5 min each), the stained cells were mounted on glass slides and visualized using a Carl Zeiss LSM710 confocal laser scanning microscope (Carl Zeiss, Jena, Germany). Primary antibodies: anti-V5 epitope (#R960-25, Invitrogen), anti-HA epitope (#11867423001, Roche), anti-Active-CASP3 (#9661, Cell Signaling Technology, Beverly, MA), and anti-TRB3 (#2488-1, Epitomics, Burlingame, CA).

### Quantification of C163S-proCASP3-EmGFP and C163S-proCASP3-HA Localization

Preferential localization of C163S-proCASP3-EmGFP in the fixed cells was assessed as either mainly cytoplasmic (C) or mainly nuclear (N) based on their observed relative fluorescence intensity using a Carl Zeiss LSM710 confocal laser scanning microscope. In a similar way, the localization of C163S-proCASP3-HA was also assessed in artificial miRNA expressing cells that could be assumed by cocistronic expression of EmGFP. To rule out the contribution of the active CASP3 to the quantification of proCASP3 localization, we examined only morphologically normal cells (98.7% of tunicamycin-treated morphologically normal cells were active CASP3 negative; see text and Figure S5).

### Live Imaging Analysis

Transfected HeLa cells grown on a glass bottom dish were set in the culture environment (37°C, 5% CO_2_) of microscope after treatment with a given agent. Images of EmGFP-fusion protein positive and DsRed positive cells, respectively, were acquired using the Carl Zeiss LSM710 confocal laser scanning microscope equipped with LSM 7 live module. Time-lapse images were taken with a 63× plan apochromatic objective with a numerical aperture of 1.4. Each frame of time-lapse images was acquired every 5 minutes (Movie S1) or 10 minutes (Figure 4C) for the indicated times. Movie S1 was shown at 10 frames/sec.

### Statistics

Data shown are mean ±S.D. Student’s t test was used to determine the significance of differences. *P* values <0.05 were considered to be statistically significant.

## Supporting Information

Figure S1 **Sequence alignment analysis of TRB3 homologs.** Organisms, more than 50% amino acid identity to human TRB3, were selected in this analysis. The result showed that TRB3 caspase-cleavage site is highly conserved. Although, homology of *Danio rerio* TRB3 is relatively low (52.9%), it should be noted that the P4-P1 substrate recognition motif (VVPD) is completely conserved. This raises a possibility that TRB3 is cleaved in various species and the cleavage has biological significance. Sequence alignment was carried out using the Geneious software. Organism, Accession number: Homo sapiens, AAH27484; Pongo abelii, XP_002830205; Macaca mulatta, XP_002798226; Callithrix jacchus, XP_002747457; Canis lupus familiaris, XP_542943; Ailuropoda melanoleuca, XP_002925440; Bos taurus, NP_001069571; Oryctolagus cuniculus, XP_002710903; Mus musculus, NP_780302; Rattus norvegicus, NP_653356; Danio rerio, NP_998034
(PDF)

Figure S2 **TNFα/CHX- and anti-Fas antibody-induced cell death was strongly inhibited by the caspase inhibitor z-VAD-FMK.** (A, B) Twenty-four hours after transfection with the V5-WT-TRB3 expression plasmid, HeLa (A) and Jurkat (B) cells were treated with TNFα (20 ng/mL)/CHX (100 µM) for 4 hr and anti-Fas antibody (125 ng/mL) for 6 hr, respectively, in the absence or presence of z-VAD-FMK (100 µM). The resulting dead cells were counted by trypan blue staining. Error bars indicate mean ±SD of three independent experiments.
(PDF)

Figure S3 **Cleavage of endogenous TRB3 in apoptotic cells.** HeLa cells were treated with tunicamycin (5 µM) for 8 hr, and then treated with TNFα/CHX in the absence or presence of z-VAD-FMK (100 µM) for 3 hr. DMSO was used as a treatment control. The cell lysates were subjected to immunoblot analysis using anti-TRB3 antibody. Cleaved PARP (#9541, Cell Signaling Technology) is a marker of apoptosis. α-Tubulin was used as an internal control.
(PDF)

Figure S4 **Tunicamycin-induced cell death was strongly inhibited by the caspase inhibitor z-VAD-FMK.** Twenty-four hours after transfection with the control vector, HeLa cells were treated with tunicamycin for 36 hr in the absence or presence of z-VAD-FMK. The resulting dead cells were counted by trypan blue staining. Error bars indicate mean ±SD of three independent experiments.
(PDF)

Figure S5 **Active CASP3 was hardly detected in tunicamycin-treated morphologically normal cells.** HeLa cells grown on coverslips were treated with tunicamycin for 8 hr. The fixed cells were stained with anti-Active-CASP3 antibody (red), and counterstained with DAPI (blue) and Alexa 488-conjugated phalloidin (green) to visualize the nuclei and cell morphology, respectively. 100 cells were assessed in three independent experiments, and 98.7% of tunicamycin-treated morphologically normal cells were active CASP3 negative. Scale bars = 20 µm.
(PDF)

Figure S6 **Nuclear translocation efficiency of proCASP3 mediated by D338A-TRB3 was almost same as that by WT-TRB3.** (A) HeLa cells grown on coverslips were cotransfected with the EmGFP-tagged inactive proCASP3 mutant (C163S-proCASP3-EmGFP) and V5-D338A-TRB3 expression plasmids, and then incubated for 24 hr. The fixed cells were stained with anti-V5 antibody (red) and counterstained with DAPI (blue) to visualize the nuclei. Scale bars = 20 µm. (B) Twenty-four hours after transfection, HeLa cells grown on coverslips were fixed, and then stained as described above. Localization of C163S-proCASP3-EmGFP in cells that are also expressing TRB3 from the respective plasmid was quantified as cytoplasmic, mainly cytoplasmic or cytoplasmic equal to nuclear (C, C>N, C = N), or as nuclear or mainly nuclear (N, N>C). Over 30 cells were assessed in three independent experiments. Error bar: mean ±SD.
(PDF)

Figure S7 **The localization of C163S-proCASP3-HA.** HeLa cells grown on coverslips were transfected with the C163S-proCASP3-HA expression plasmid. After 24 hr, the cells were incubated with or without tunicamycin for 8 hr. The localization of C163S-proCASP3-HA was observed by immunofluorescence staining with an anti-HA antibody (red). Inner panel was merged with DAPI (blue) to visualize the nuclei. Scale bars = 20 µm.
(PDF)

Figure S8 **Artificial miRNAs targeting TRB3 mRNA specifically suppress endogenous TRB3 expression.** HeLa cells grown on coverslips were transfected with the indicated artificial miRNA expression plasmid that enable identify the miRNA expressing cells by cocistronic expression of EmGFP. After 24 hr, the cells were treated with tunicamycin for 8 hr, and then fixed. Endogenous TRB3 and nuclei were visualized by immunofluorescence staining with an anti-TRB3 antibody (red) and DAPI (blue) respectively. Scale bars = 20 µm. miR-NC denotes negative control miRNA.
(PDF)

Figure S9 **Expression of V5-ΔC20-TRB3 did not affect CASP3/7 activation and apoptosis.** (A) HeLa cells were transfected with the V5-ΔC20-TRB3 expression plasmid. Twenty-four hours after, cells were treated with TNFα/CHX for 4 hr. CASP3/7 activity was measured in V5-ΔC20-TRB3 expressing HeLa cells as described in the legend of Figure 2C. Error bars indicate mean ±SD of three independent experiments. (B) Twenty-four hours after transfection with the V5-ΔC20-TRB3 expression plasmid or control vector, HeLa cells were treated with TNFα/CHX for 4 hr. The resulting dead cells were counted by trypan blue staining. Error bars indicate mean ±SD of three independent experiments.
(PDF)

Figure S10 **The effect of CASP2 on TRB3 under ER stress condition.** Twenty-four hours after transfection with the indicated V5-TRB3 expression plasmid or control vector, HeLa cells were treated with tunicamycin in the absence or presence of CASP2 inhibitor z-VDVAD-FMK (10 µM) (BioVision) for 24 hr. The cell lysates were subjected to immunoblot analysis using anti-V5 antibody (A). Alternatively, the resulting dead cells were counted by trypan blue staining (B). Error bars indicate mean ±SD of four independent experiments. *P<0.005, **P<0.001.
(PDF)

Figure S11 **The effect of TRB3 on ER stress independent apoptosis induced by staurosporine.** Twenty-four hours after transfection with the indicated V5-TRB3 expression plasmid or control vector, HeLa cells were treated with staurosporine (STS) (50 nM) (Wako Pure Chemical Industries, Osaka, Japan) for 24 hr. The resulting dead cells were counted by trypan blue staining (A). Error bars indicate mean ±SD of four independent experiments. *P<0.005. Alternatively, the cell lysates were subjected to immunoblot analysis using anti-V5 antibody. Tunicamycin (5 µM) (B) or CASP9 inhibitor Ac-LEHD-CHO (10 µM) (Calbiochem) (C) was used for a comparison analysis of TRB3 cleavage.
(PDF)

Figure S12 **A hypothetical model for the stress response mechanism of TRB3.**
(PDF)

Movie S1 **Nuclear distribution of proCASP3 is not critical for the execution of apoptosis.**
(MOV)
