# Supplementary material for: Stress-Inducible Caspase Substrate TRB3 Promotes Nuclear Translocation of Procaspase-3
Source: PLoS One. 2012 Aug 9;7(8):e42721. doi: 10.1371/journal.pone.0042721 (PMC3415431; doi:10.1371/journal.pone.0042721)
Supplement: Figure S1 — Sequence alignment analysis of TRB3 homologs. Organisms, more than 50% amino acid identity to human TRB3, were selected in this analysis. The result showed that TRB3 caspase-cleavage site is highly conserved. Although, homology of Danio rerio TRB3 is relatively low (52.9%), it should be noted that the P4-P1 substrate recognition motif (VVPD) is completely conserved. This raises a possibility that TRB3 is cleaved in various species and the cleavage has biological significance. Sequence alignment was carried out using the Geneious software. Organism, Accession number: Homo sapiens, AAH27484; Pongo abelii, XP_002830205; Macaca mulatta, XP_002798226; Callithrix jacchus, XP_002747457; Canis lupus familiaris, XP_542943; Ailuropoda melanoleuca, XP_002925440; Bos taurus, NP_001069571; Oryctolagus cuniculus, XP_002710903; Mus musculus, NP_780302; Rattus norvegicus, NP_653356; Danio rerio, NP_998034 (PDF) [file pone.0042721.s001.pdf]

Figure S1

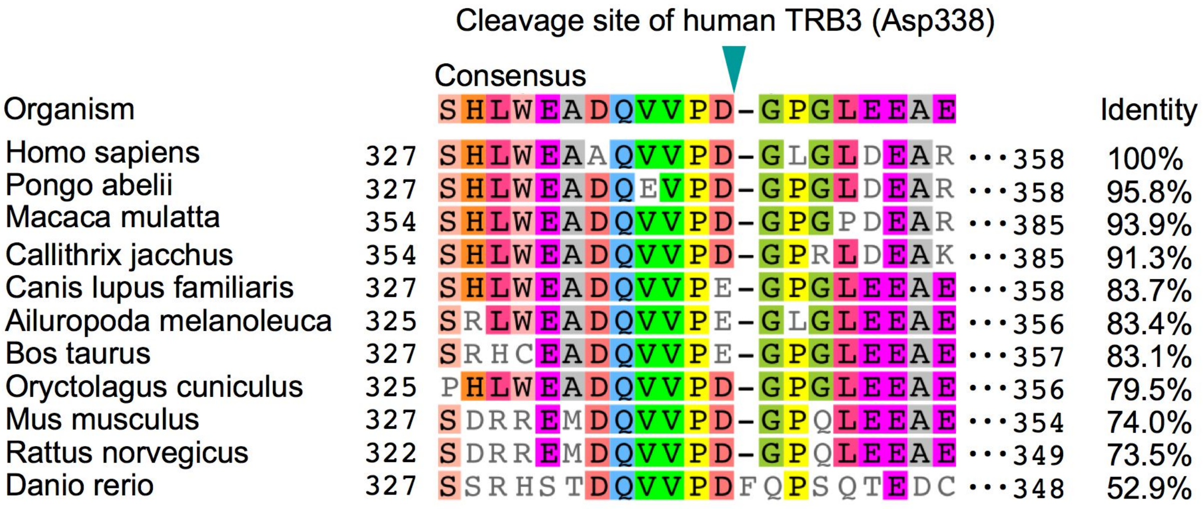
Cleavage site of human TRB3 (Asp338)
Consensus
Organism
SHLWEADQVVPD-GPGLEEAE
Identity
Homo sapiens 327 SHLWEAAQVVPD-GLGLDEAR ···358 100%
Pongo abelii 327 SHLWEADQEVPD-GPGLDEAR ···358 95.8%
Macaca mulatta 354 SHLWEADQVVPD-GPGPDEAR ···385 93.9%
Callithrix jacchus 354 SHLWEADQVVPD-GPRLDEAK ···385 91.3%
Canis lupus familiaris 327 SHLWEADQVVPE-GPGLEEAE ···358 83.7%
Ailuropoda melanoleuca 325 SRLWEADQVVPE-GLGLEEAE ···356 83.4%
Bos taurus 327 SRHCEADQVVPE-GPGLEEAE ···357 83.1%
Oryctolagus cuniculus 325 PHLWEADQVVPD-GPGLEEAE ···356 79.5%
Mus musculus 327 SDRREMDQVVPD-GPQLEEAE ···354 74.0%
Rattus norvegicus 322 SDRREMDQVVPD-GPQLEEAE ···349 73.5%
Danio rerio 327 SSRHSTDQVVPDFQPSQTEDC ···348 52.9%
