# Supplementary figures and images for: Stress-Inducible Caspase Substrate TRB3 Promotes Nuclear Translocation of Procaspase-3
Source: PLoS One. 2012 Aug 9;7(8):e42721. doi: 10.1371/journal.pone.0042721 (PMC3415431; doi:10.1371/journal.pone.0042721)

Figure S2

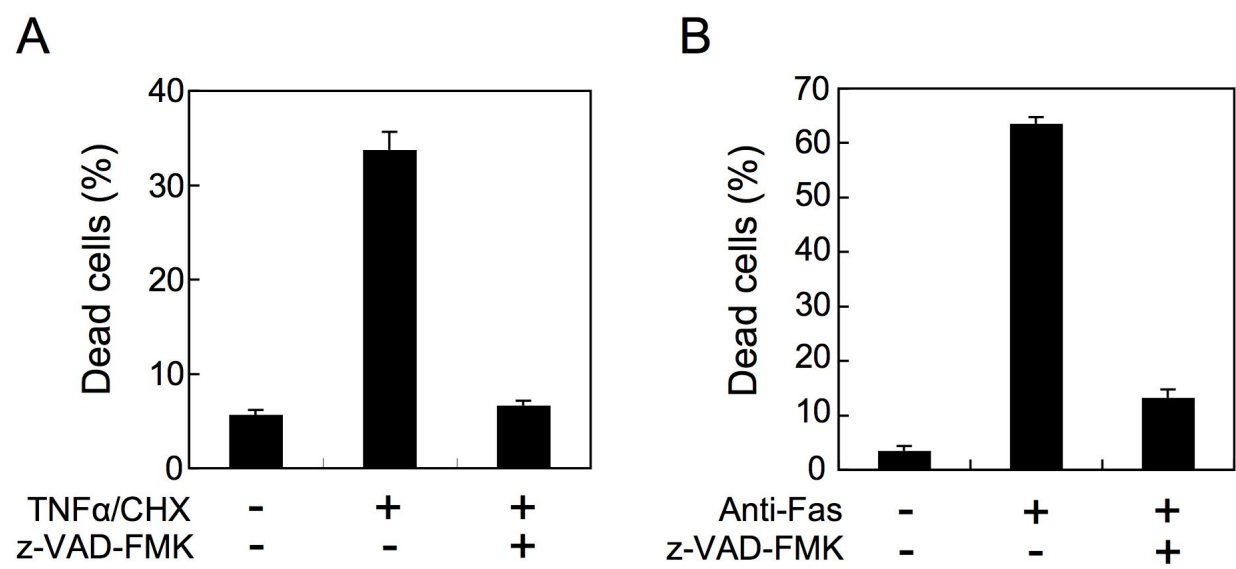

Supplement: Figure S2 — TNFα/CHX- and anti-Fas antibody-induced cell death was strongly inhibited by the caspase inhibitor z-VAD-FMK. (A, B) Twenty-four hours after transfection with the V5-WT-TRB3 expression plasmid, HeLa (A) and Jurkat (B) cells were treated with TNFα (20 ng/mL)/CHX (100 µM) for 4 hr and anti-Fas antibody (125 ng/mL) for 6 hr, respectively, in the absence or presence of z-VAD-FMK (100 µM). The resulting dead cells were counted by trypan blue staining. Error bars indicate mean ±SD of three independent experiments. (PDF) [file pone.0042721.s002.pdf]

# Figure S3

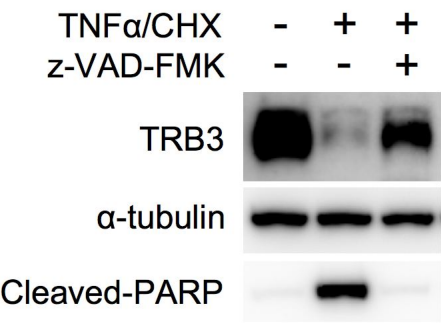

Supplement: Figure S3 — Cleavage of endogenous TRB3 in apoptotic cells. HeLa cells were treated with tunicamycin (5 µM) for 8 hr, and then treated with TNFα/CHX in the absence or presence of z-VAD-FMK (100 µM) for 3 hr. DMSO was used as a treatment control. The cell lysates were subjected to immunoblot analysis using anti-TRB3 antibody. Cleaved PARP (#9541, Cell Signaling Technology) is a marker of apoptosis. α-Tubulin was used as an internal control. (PDF) [file pone.0042721.s003.pdf]

Figure S4

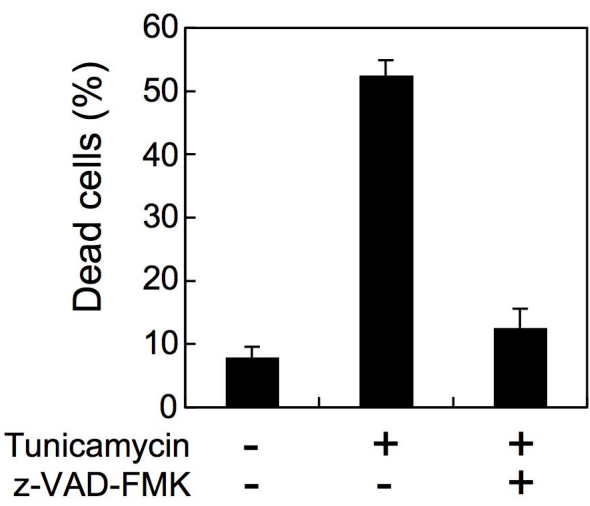

Supplement: Figure S4 — Tunicamycin-induced cell death was strongly inhibited by the caspase inhibitor z-VAD-FMK. Twenty-four hours after transfection with the control vector, HeLa cells were treated with tunicamycin for 36 hr in the absence or presence of z-VAD-FMK. The resulting dead cells were counted by trypan blue staining. Error bars indicate mean ±SD of three independent experiments. (PDF) [file pone.0042721.s004.pdf]

Figure S5

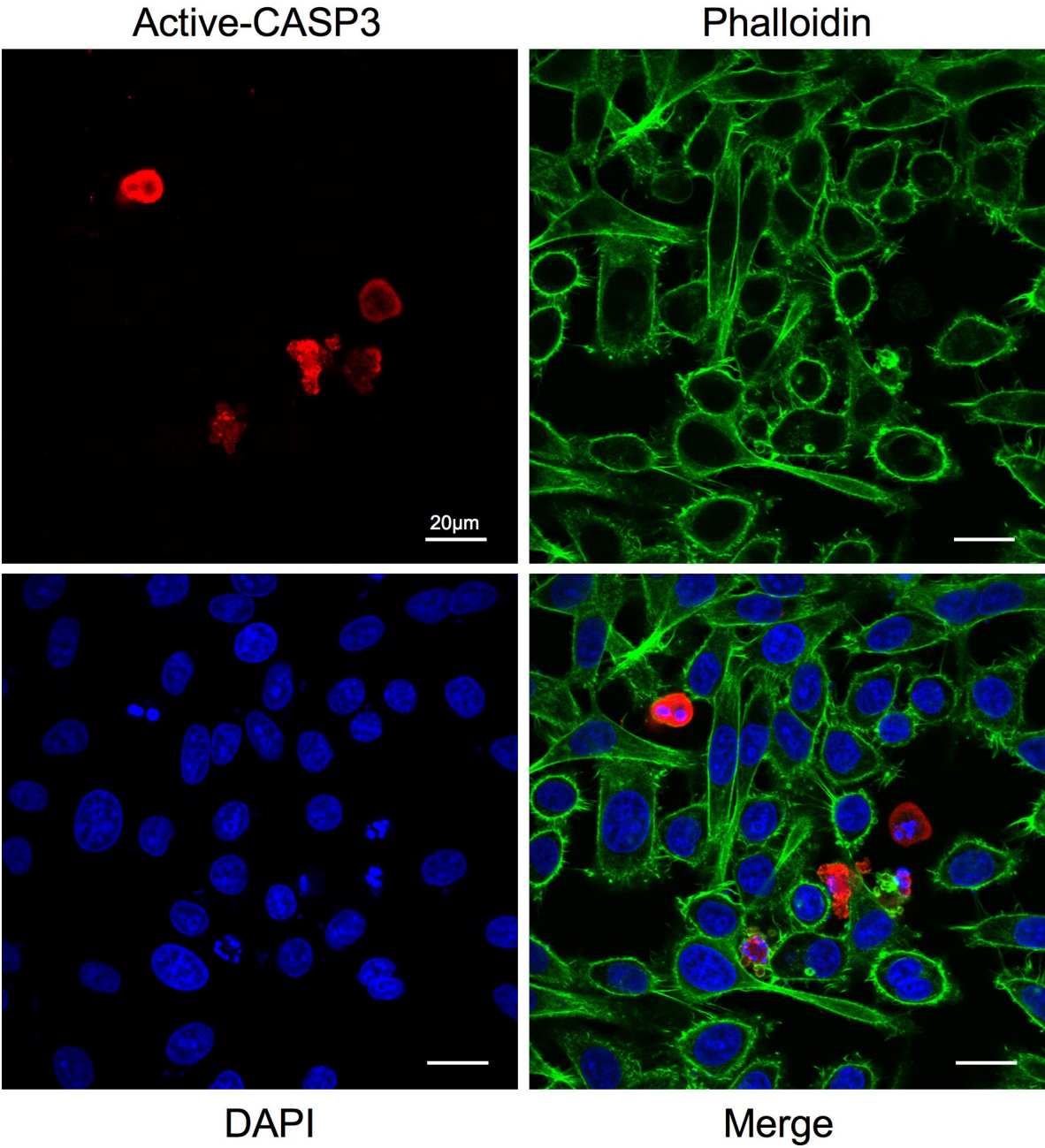

Supplement: Figure S5 — Active CASP3 was hardly detected in tunicamycin-treated morphologically normal cells. HeLa cells grown on coverslips were treated with tunicamycin for 8 hr. The fixed cells were stained with anti-Active-CASP3 antibody (red), and counterstained with DAPI (blue) and Alexa 488-conjugated phalloidin (green) to visualize the nuclei and cell morphology, respectively. 100 cells were assessed in three independent experiments, and 98.7% of tunicamycin-treated morphologically normal cells were active CASP3 negative. Scale bars = 20 µm. (PDF) [file pone.0042721.s005.pdf]

# Figure S6

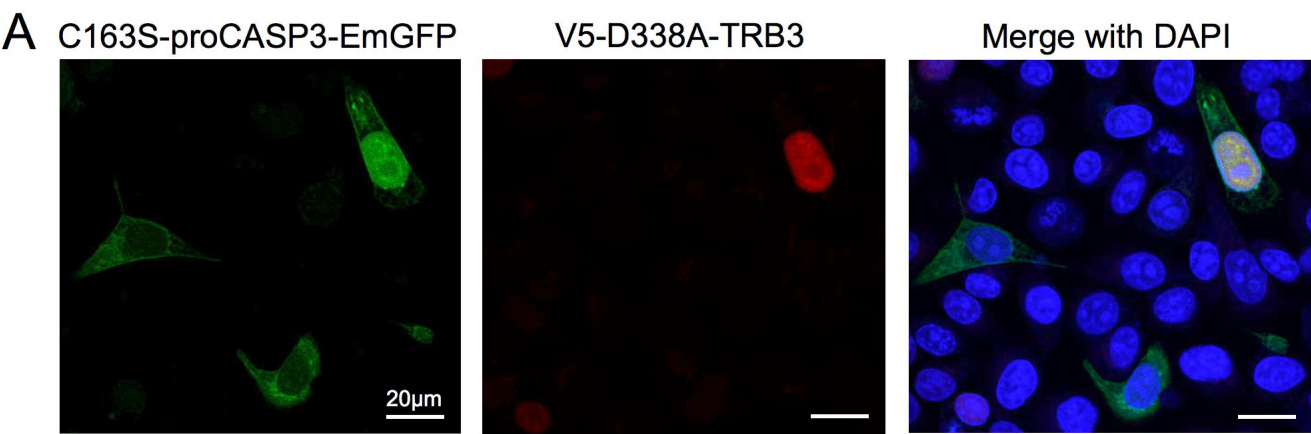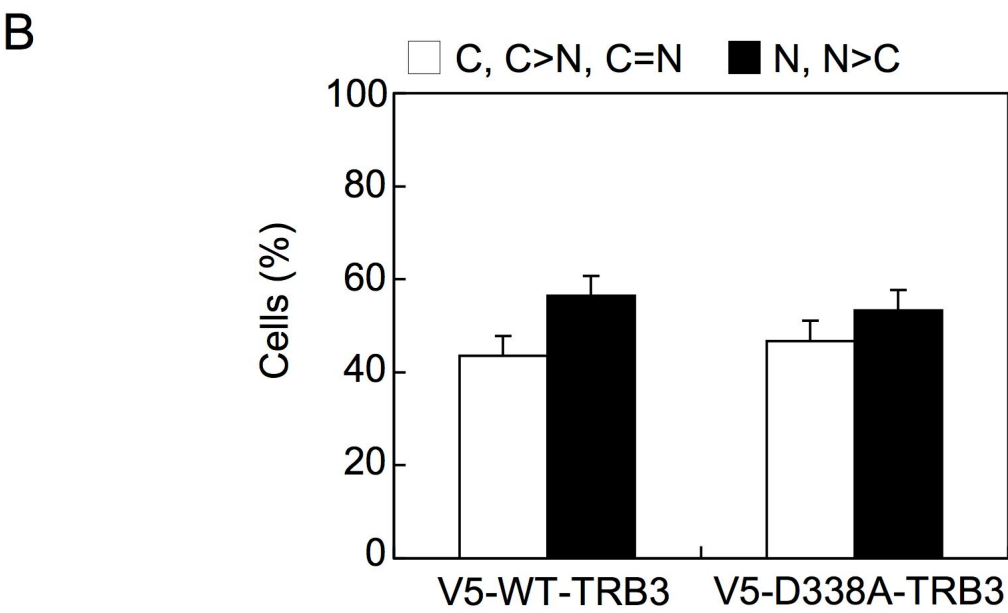

Supplement: Figure S6 — Nuclear translocation efficiency of proCASP3 mediated by D338A-TRB3 was almost same as that by WT-TRB3. (A) HeLa cells grown on coverslips were cotransfected with the EmGFP-tagged inactive proCASP3 mutant (C163S-proCASP3-EmGFP) and V5-D338A-TRB3 expression plasmids, and then incubated for 24 hr. The fixed cells were stained with anti-V5 antibody (red) and counterstained with DAPI (blue) to visualize the nuclei. Scale bars = 20 µm. (B) Twenty-four hours after transfection, HeLa cells grown on coverslips were fixed, and then stained as described above. Localization of C163S-proCASP3-EmGFP in cells that are also expressing TRB3 from the respective plasmid was quantified as cytoplasmic, mainly cytoplasmic or cytoplasmic equal to nuclear (C, C>N, C = N), or as nuclear or mainly nuclear (N, N>C). Over 30 cells were assessed in three independent experiments. Error bar: mean ±SD. (PDF) [file pone.0042721.s006.pdf]

Figure S7

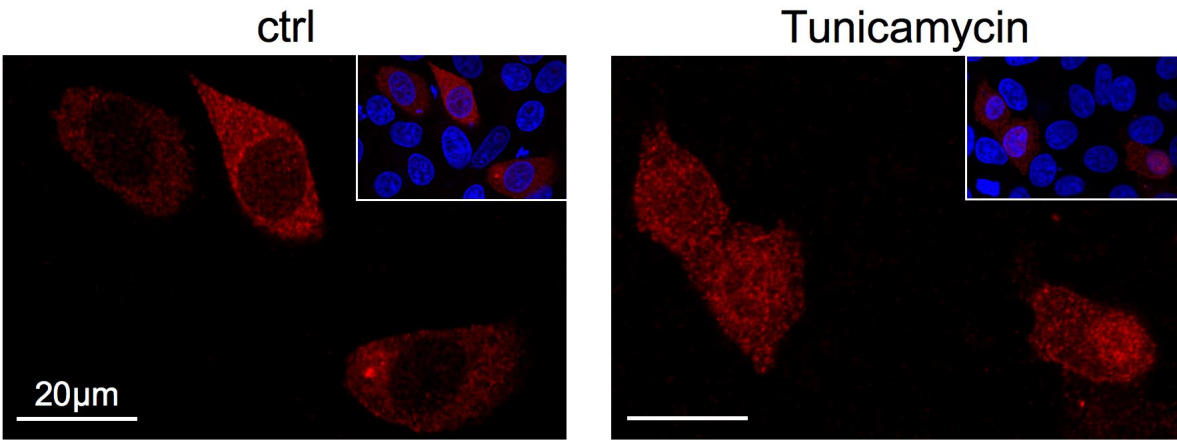

Supplement: Figure S7 — The localization of C163S-proCASP3-HA. HeLa cells grown on coverslips were transfected with the C163S-proCASP3-HA expression plasmid. After 24 hr, the cells were incubated with or without tunicamycin for 8 hr. The localization of C163S-proCASP3-HA was observed by immunofluorescence staining with an anti-HA antibody (red). Inner panel was merged with DAPI (blue) to visualize the nuclei. Scale bars = 20 µm. (PDF) [file pone.0042721.s007.pdf]

Figure S8

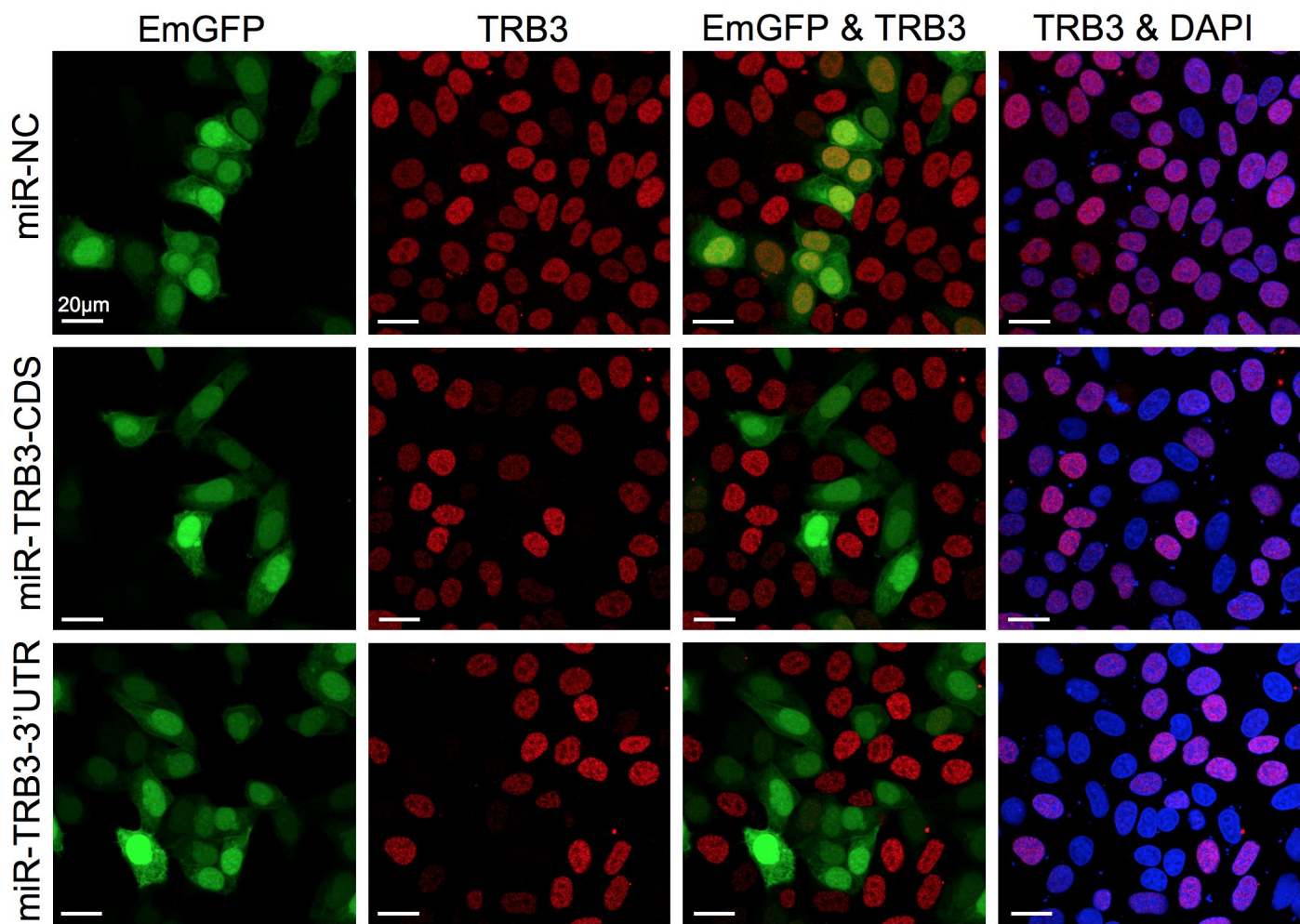

Supplement: Figure S8 — Artificial miRNAs targeting TRB3 mRNA specifically suppress endogenous TRB3 expression. HeLa cells grown on coverslips were transfected with the indicated artificial miRNA expression plasmid that enable identify the miRNA expressing cells by cocistronic expression of EmGFP. After 24 hr, the cells were treated with tunicamycin for 8 hr, and then fixed. Endogenous TRB3 and nuclei were visualized by immunofluorescence staining with an anti-TRB3 antibody (red) and DAPI (blue) respectively. Scale bars = 20 µm. miR-NC denotes negative control miRNA. (PDF) [file pone.0042721.s008.pdf]

Figure S9

A

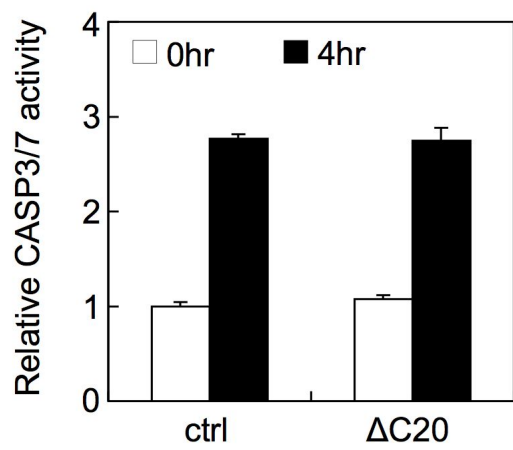

B

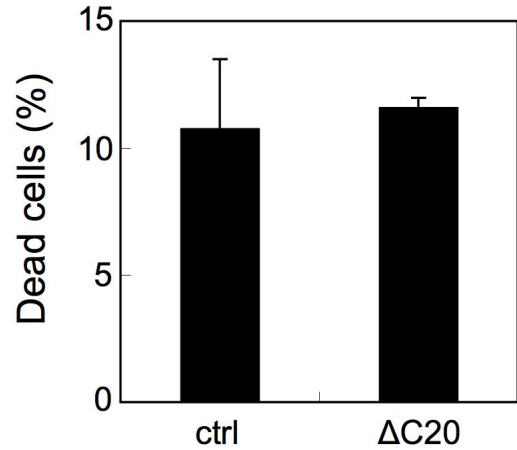

Supplement: Figure S9 — Expression of V5-ΔC20-TRB3 did not affect CASP3/7 activation and apoptosis. (A) HeLa cells were transfected with the V5-ΔC20-TRB3 expression plasmid. Twenty-four hours after, cells were treated with TNFα/CHX for 4 hr. CASP3/7 activity was measured in V5-ΔC20-TRB3 expressing HeLa cells as described in the legend of Figure 2C. Error bars indicate mean ±SD of three independent experiments. (B) Twenty-four hours after transfection with the V5-ΔC20-TRB3 expression plasmid or control vector, HeLa cells were treated with TNFα/CHX for 4 hr. The resulting dead cells were counted by trypan blue staining. Error bars indicate mean ±SD of three independent experiments. (PDF) [file pone.0042721.s009.pdf]

# Figure S10

A

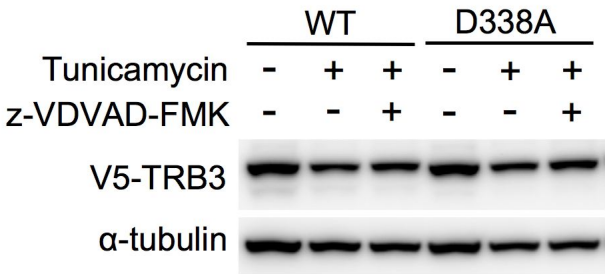

B

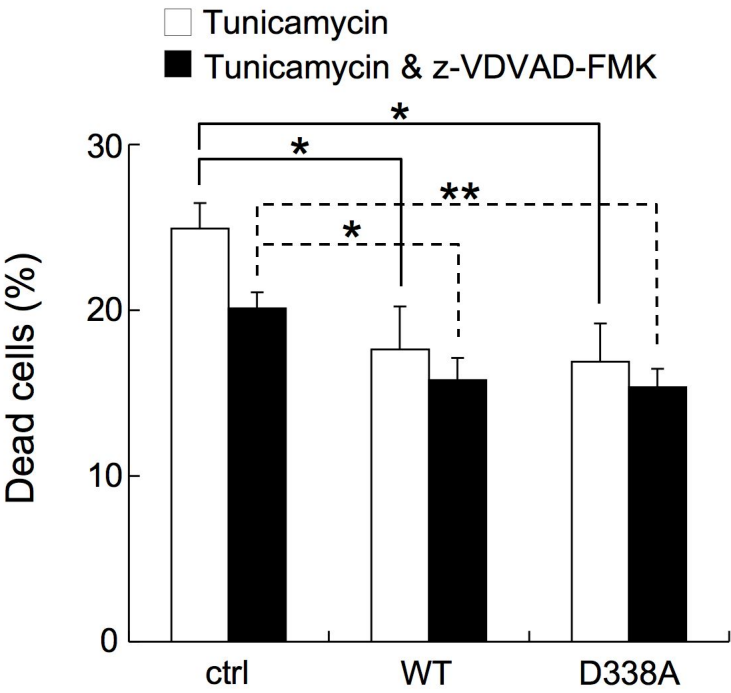

Supplement: Figure S10 — The effect of CASP2 on TRB3 under ER stress condition. Twenty-four hours after transfection with the indicated V5-TRB3 expression plasmid or control vector, HeLa cells were treated with tunicamycin in the absence or presence of CASP2 inhibitor z-VDVAD-FMK (10 µM) (BioVision) for 24 hr. The cell lysates were subjected to immunoblot analysis using anti-V5 antibody (A). Alternatively, the resulting dead cells were counted by trypan blue staining (B). Error bars indicate mean ±SD of four independent experiments. *P<0.005, **P<0.001. (PDF) [file pone.0042721.s010.pdf]

Figure S11

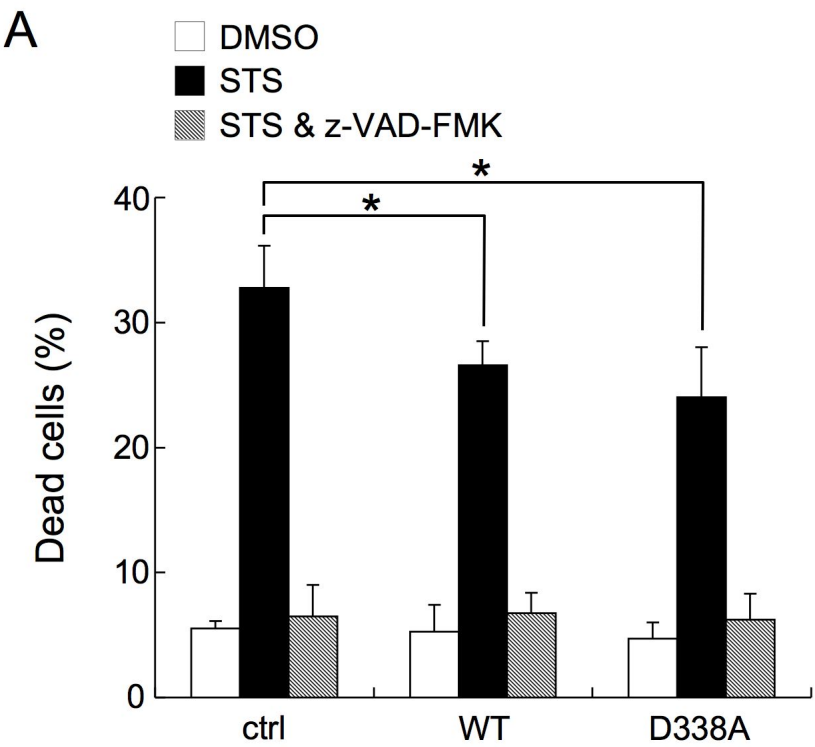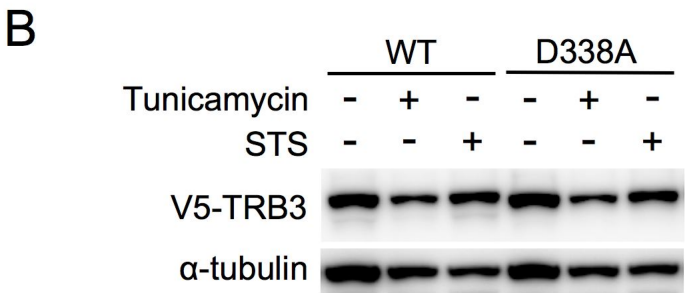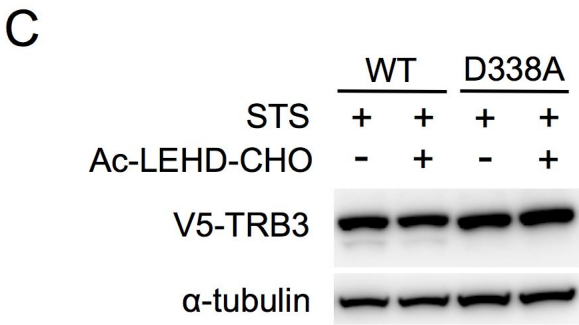

Supplement: Figure S11 — The effect of TRB3 on ER stress independent apoptosis induced by staurosporine. Twenty-four hours after transfection with the indicated V5-TRB3 expression plasmid or control vector, HeLa cells were treated with staurosporine (STS) (50 nM) (Wako Pure Chemical Industries, Osaka, Japan) for 24 hr. The resulting dead cells were counted by trypan blue staining (A). Error bars indicate mean ±SD of four independent experiments. *P<0.005. Alternatively, the cell lysates were subjected to immunoblot analysis using anti-V5 antibody. Tunicamycin (5 µM) (B) or CASP9 inhibitor Ac-LEHD-CHO (10 µM) (Calbiochem) (C) was used for a comparison analysis of TRB3 cleavage. (PDF) [file pone.0042721.s011.pdf]

**Figure S12**

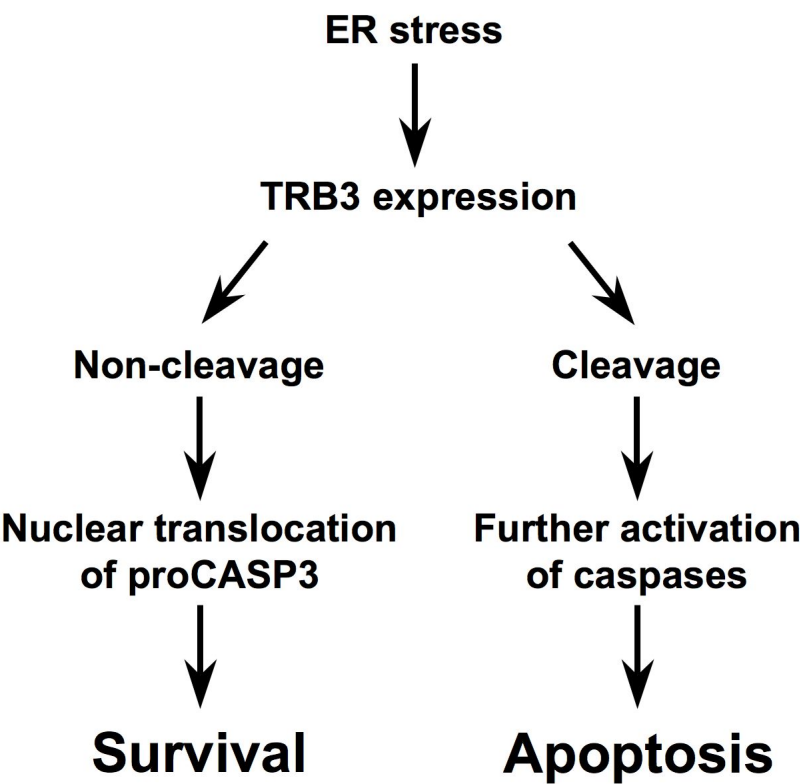

Supplement: Figure S12 — A hypothetical model for the stress response mechanism of TRB3. (PDF) [file pone.0042721.s012.pdf]
